# Case fatality ratios for serious emergency conditions in the Republic of Ireland: a longitudinal investigation of trends over the period 2002–2014 using joinpoint analysis

**DOI:** 10.1186/s12913-018-3260-1

**Published:** 2018-06-19

**Authors:** Brenda Lynch, Anthony P. Fitzgerald, Paul Corcoran, Orla Healy, Claire Buckley, Conor Foley, John Browne

**Affiliations:** 1 University College Cork, School of Public Health, 4th Floor, Western Gateway Building, Western Road, Cork City, Ireland; 2 Health Service Executive, South/South West Hospital Group, Ernville, Western Road, Cork, Ireland

**Keywords:** Reconfiguration, Emergency care, Health systems, Regional variations

## Abstract

**Background:**

In the past decade, the Republic of Ireland has undertaken significant reconfiguration programmes to improve emergency services. During this time the public healthcare system experienced a large real decrease in resources. This study assesses national and regional population outcomes over the period 2002–2014, and whether changes coincide with system reconfiguration and the financial restrictions imposed by the 2008 recession.

**Methods:**

Case fatality ratios (CFRs) were constructed for emergency conditions for 2002–2014. Total emergency conditions and individual condition trends were analysed nationally using joinpoint analysis. National results informed the investigation of trends at a regional and county level using an inverse standard error weighted generalised linear model with a log link to construct funnel plots. County-level CFRs were compared for the first and last 3 years of the period to further investigate the changes to county results over the 13 year period, specifically in comparison to the national-level CFR.

**Results:**

Nationally, there was an annual fall in CFRs (2.1%). The decline was faster from 2002 to 2007 (annual percentage change = − 3.4; 95% CI-4.4, − 2.4), compared to 2007–2014 (annual percentage change = − 1.2; 95% CI -1.9, − 0.5). The South-East had a lower rate of decrease and the West had a higher rate. Cross sectional analysis of two periods (2002–2004 and 2012–2014) showed high consistency in the counties performance relative to the national CFR in both periods.

**Conclusion:**

Change in the national trend coincided with the onset of economic stress on the public health system. Attributing the decline in CFR improvement to economic factors is weakened by the uneven nature of the trend change. No distinct pattern of change was identified among regions which underwent substantial reconfiguration of emergency services.

**Electronic supplementary material:**

The online version of this article (10.1186/s12913-018-3260-1) contains supplementary material, which is available to authorized users.

## Background

Conditions requiring emergency medical treatment are significant contributors to global mortality. Ischaemic heart disease, including myocardial infarction, accounted for almost 16% of total deaths in 2015 (8.9 million deaths) [1]. Ischaemic and haemorrhagic stroke were the second largest cause of total deaths at 11.3% (6.3 million deaths) [1]. A further 8.5% of total deaths (4.7 million deaths) were due to external injuries [1].

Outcomes from serious emergency conditions in the Republic of Ireland (Ireland hereafter) are broadly similar to other Organisation for Economic Co-operation and Development (OECD) countries. Analysis of hospital mortality in Ireland found significant reductions in deaths from acute myocardial infarction, heart failure and ischaemic stroke between 2005 and 2015 [2]. The most recent 2011 analysis by the OECD found in-hospital mortality for ischaemic stroke in Ireland (9.9 deaths per 100 admissions) was higher than the OECD average of 8.5, but was lower than the OECD average (7.9) for myocardial infarction (at 6.8 deaths per 100 admissions) [3]. In Ireland, no previous research of total case fatality for serious emergency conditions has included patients who die outside of hospital. Also, no research has been performed on outcomes for residents of different geographical regions; the focus continues to be on hospital level outcomes [4–6].

Similar to other countries, emergency care services have been centralised to varying degrees across Ireland in the last decade [7–10]. Common features include reducing access to emergency departments in smaller hospitals, centralising specialist emergency care at a ‘hub’ hospital, and integrating ambulance and general practice referral protocols for given conditions. This reconfiguration has largely occurred in southern and western regions, which are also the most rural. These changes coincided with the establishment of many international best practice recommendations, clinical programmes and guidelines for the treatment of emergency conditions including stroke, acute myocardial infarction (AMI) and trauma [11–14].

Centralising emergency care services has proven controversial. While patients are theoretically transported directly to services appropriate to the severity of their condition, longer journey times exacerbate underlying risks associated with rural areas [15]. International studies highlight the geographical variation in survival from emergency conditions; there is a greater risk of poorer outcomes due to distance from acute services and the existence of an older more socioeconomically disadvantaged population in rural areas [16–18]. The interactions between rurality and deprivation contribute to the complexity of understanding any variation in total mortality [19]. However, the majority of studies consistently emphasis the impact of hospital closures on in-hospital mortality. The continued focus on this outcome may conceal potential increases to out-of-hospital deaths caused by increased travel times.

In Ireland, the changes to services brought about by reconfiguration have not happened in isolation. From 2008, the country experienced an economic recession which resulted in substantial decreases in funding and staff across the public healthcare service. It is estimated that public funding for healthcare was reduced by 22% over the period 2009–2013 and staffing of public services fell by 10% from a peak level in 2007 [20].

Within this paper, case fatality for a number of serious emergency conditions in Ireland over the period 2002–2014 is investigated at a national, regional and county level. The aim is to describe trends in case fatality and establish if, and how, any changes coincide with reconfiguration events and the timing of the economic recession.

## Methods

### Study area and context

**Fig. 1:**
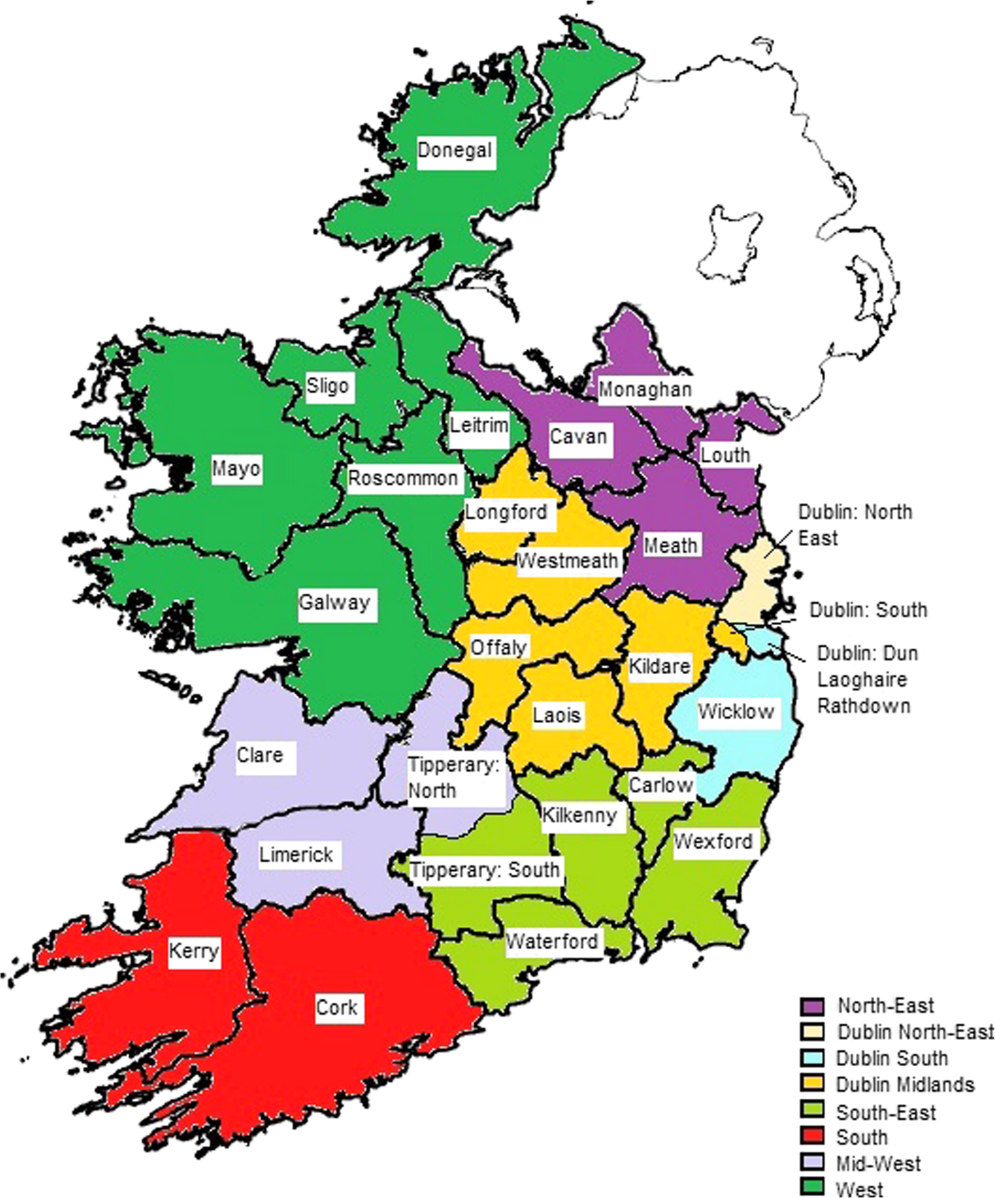
Ireland: Regions and counties

The Republic of Ireland is an island of 70.2 thousand km^2^ on the west of Europe with a population of 4.8 million [21]. It is divided into 26 counties, which for the analysis of emergency care reconfiguration may be grouped into eight regions based on hospital networks identified by the organisation that delivers public healthcare services in Ireland (Fig. 1) [22].

Regional characteristics and reconfiguration of services are presented in online Additional file 1. Two regions (South and Mid-West) have implemented significant reconfiguration of urgent and emergency care over the period 2012–2014. Four regions (West, North-East, South-East and Dublin-South) have introduced some reconfiguration measures since 2006, but these do not cover all services. The two remaining regions (Dublin Midlands and Dublin North-East) have undertaken no major changes since 2006. Emergency departments were eliminated in four largely rural counties: Clare (2009), Tipperary North (2009), Roscommon (2011) and Monaghan (2009). Four other rural counties did not have an emergency department throughout the study period: Leitrim, Wicklow, Carlow and Longford.

### Data sources

We considered 16 serious emergency conditions, derived from consensus work carried out in the UK, for which the risk of death could be reduced by a well performing emergency care system [23]. The conditions were grouped into three categories; stroke, acute myocardial infarction/cardiac arrest, and ‘other’ (see Table 1 and Additional file 2).

**Table 1:** Basket of Emergency Conditions

**1**	**Stroke**
**2**	**Acute Myocardial Infarction and Cardiac Arrest (AMI and CA)**
**3**	**Other** Acute heart failure, anaphylaxis, asphyxiation, asthma, falls under 75, fractured neck of femur, meningitis, pregnancy, road traffic accident, ruptured aortic, self-harm, septic shock, serious head injury

The incidences of deaths from the selected emergency conditions are available from the Irish Central Statistics Office (see Additional file 3). The incidence of hospital admissions for these conditions is available from the Irish Hospital In-Patient Enquiry (HIPE) admissions system.

The regions and constituent counties analysed are outlined in Fig. 1. Due to concerns regarding the completeness of HIPE data for Roscommon over the period 2011–12, this county has been omitted from all analyses for these years.

Historically, Dublin County has been divided into three sections with respect to emergency care delivery, Dublin North-East, Dublin-South and Dublin Midlands. However, mortality and admissions data is not available at a sub-county level. Therefore, CFRs for Dublin were analysed as a whole and reported independently. As a consequence we only present data for the Midlands part of the Dublin-Midlands region (i.e. counties Kildare, Laois, Offaly, Westmeath and Longford) and for the Wicklow part of the Dublin-South region.

Results produced at a county level also allow for comparisons with routinely collected measures from other administrative data sources.

### Statistical analysis

#### National case fatality ratios (CFRs)

The primary outcome of interest is case fatality ratios (CFR). Annual CFRs from 2002 to 2014 inclusive were calculated. 2002 was chosen as the initial year of analysis as it is the first year that HIPE allows restriction by admission type i.e. emergency admission.

CFRs were calculated by dividing deaths due to the relevant conditions by an estimate of the case incidence. Case incidence was constructed by adding the number of patients who were admitted to a public hospital with one of the emergency conditions and discharged alive after at least a 2 day length of stay, to the number of deaths from that condition (see Additional file 4) [23].

CFRs have been found to be dependent on condition and age, but not sex [23]. Therefore, all CFRs were directly standardised using estimates of the national population’s age and case-mix composition in 2014. With 16 conditions and 18 age groups, the age-condition specific CFRs in some regions were small and often zero (see Additional files 3 and 4) [23]. Therefore, conditions were grouped, and ages reduced to those under 65 and then 5 year age groups to 85+ to allow for meaningful direct standardisation. To adjust for case-mix, the national case incidence rate for each of the condition categories, by age group, were generated and these weights were multiplied by each region and county’s case fatality rate.

#### Joinpoint analysis of national trends

Joinpoint analysis was conducted on the annual adjusted national CFR observations from 2002 to 2014. This identifies possible change-points where a significant change in the linear trend in national case fatality on a log scale is detected over the study period [24]. The analysis was conducted using the software developed by the Surveillance Research Program Version 4.2.0.1 of USA National Cancer Institute.

Models with a single joinpoint were considered and the optimal piecewise linear model was compared to one with no joinpoints i.e. a straight line. To describe linear trends by period, the estimated annual percent change (APC) is computed for each trend by fitting a regression line to the natural logarithm of the rates using the calendar year as a regressor variable [24]. A negative APC signifies an annual decrease in case fatality, while a positive result denotes an increase. National results were deemed to have a statistically significant change in trend if the results from the estimated regression coefficients for the difference in the slopes had a *P* value less than 0.05.

#### Generalised linear model and funnel plot of regional and county CFR trends

Longitudinal trends in standardised CFRs were estimated at region and county level using an inverse standard error weighted generalised linear model with a log link, informed by the identified joinpoint in national trends. The inverse standard error allows for precision in comparing areas with differing case populations [25]. Models included the age and case-mix adjusted rate as the dependent variable, with area and an interaction between year and area as independent variables. Trends were compared to the national annual trend using a funnel plot with 95% limits (+/− 2 standard deviations) to identify any areas that differed significantly from the national result.

#### Cross sectional analysis of county CFRs in two time periods

A cross sectional analysis was used to compare county CFRs in two different 3 year time periods, 2002–2004, and 2012–2014. The focus on county CFRs allows specific examination of areas that had emergency department closures. Results were compared to the national CFR to establish if a county was above or below the national result in both periods, and a Spearman rank correlation of results was calculated to describe the consistency in a county’s performance over the two periods. The coefficient of variation was calculated to determine if the variance between county results had increased or decreased between the two periods. This analysis was conducted using Stata (Version 13).

## Results

### National case fatality ratios (CFRs)

Case fatality ratios were constructed for each year from 2002 to 2014 inclusive. For 2002–2004 the national annual CFR was 187 per 1000, falling to 151 per 1000 over the period 2012–2014. The national annual percentage change in national total CFRs over the period 2002–2014 was a decrease of 2.12%.

Between 2002 and 2014 the national total deaths from the selected conditions fell from 7,978 to 5,205, decreasing across all groups (see Additional file 3). Total cases also decreased from 41,645 to 35,736, again decreasing in each group (see Additional file 4).

### Joinpoint analysis of national trends

**Fig. 2:**
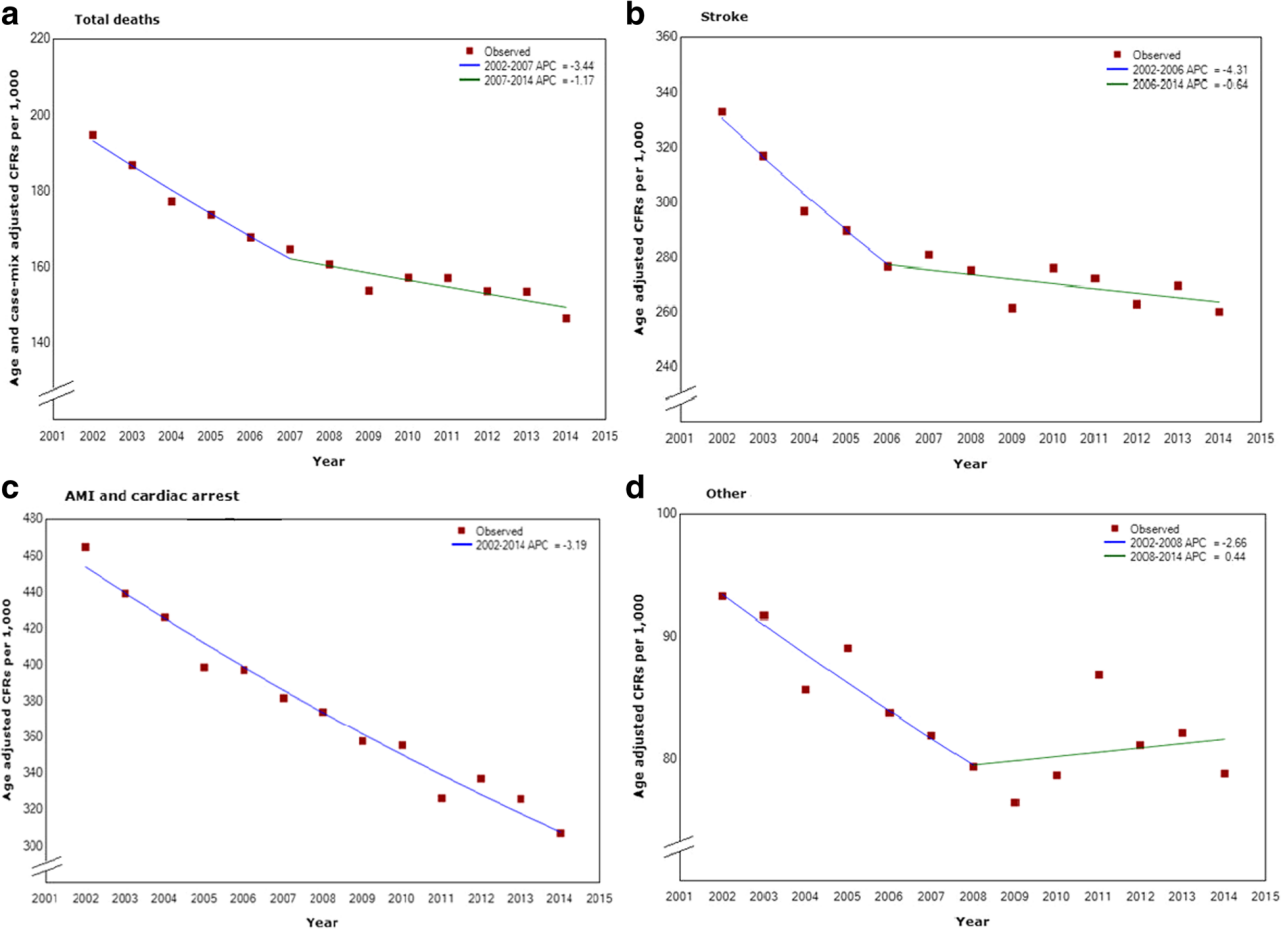
Joinpoint analyses of adjusted CFRs

Joinpoint analysis found a statistically significant change in the CFR trend for total conditions in 2007 (Fig. 2). The APC for the period 2002–2007 was − 3.4 (95% CI: -4.4, − 2.4), with the APC decreasing to − 1.2 (95% CI: -1.9, − 0.5) from 2007 to 2014.

Analysis of the individual condition groups showed a significant change in trend for stroke; from 2002 to 2006 an APC of − 4.3 (95% CI: -6.2, − 2.4) was observed, which decreased to − 0.6 (95% CI: -1.4, 0.1) from 2006 to 2014. A significant change was also seen for the ‘other’ group; from 2002 to 2008 an APC of − 2.7 (95% CI: -4.6, − 0.7) was observed, reducing to 0.4 (95% CI: -1.7, 2.6) from 2008 to 2014.

The AMI and cardiac arrest group showed a consistent downward APC of − 3.2 over the full period (95% CI: -3.5, − 2.9) (Fig. 2).

### Generalised linear model and funnel plot of regional and county CFR trends

Having identified 2007 as the relevant joinpoint in the national trend, analysis of the regional total CFR trend revealed a statistically significant downward trend from 2007 in three regions; Mid-West, West, Dublin (Table 2).

**Table 2:** Regional and County Annual Percentage Change 2007–2014 and CFRs 2002–2004 and 2012–2014

	Annual Percentage Change 2007–2014 (95% CI)	*P* |z|	CFR per 1000, 2002–2004 (95% CI)	CFR per 1000, 2012–2014 (95% CI)
Rep. of Ireland	− 1.25 (− 1.80, − 0.70)	0.000	187 (184, 189)	151 (149, 153)
Region				
Dublin	−1.56 (− 2.62, − 0.5)	0.004	180 (175, 184)	138 (134, 142)
Midlands	0.17 (− 1.34, 1.69)	0.823	192 (185, 198)	156 (150, 162)
Wicklow	− 0.82 (− 3.66, 2.02)	0.573	192 (179, 204)	154 (142, 166)
Mid-West	− 1.83 (− 3.34, − 0.33)	0.017	204 (197, 211)	168 (161, 174)
North-East	−1.4 (− 2.95, 0.15)	0.077	166 (160, 172)	141 (135, 147)
South	− 1.05 (− 2.21, 0.1)	0.073	197 (191, 202)	170 (165, 175)
South-East	0.83 (− 0.58, 2.25)	0.249	175 (170, 181)	153 (147, 158)
West	− 2.35 (− 3.44, − 1.26)	< 0.001	195 (190, 200)	150 (145, 155)
County				
Carlow	3.18 (− 0.27, 6.63)	0.071	187 (169, 205)	165 (148, 182)
Cavan	−1.91 (− 4.59, 0.78)	0.164	169 (157, 182)	148 (134, 162)
Clare	−2.07 (− 4.32, 0.19)	0.073	204 (191, 218)	163 (151, 176)
Cork	−0.58 (− 1.66, 0.5)	0.293	197 (190, 203)	164 (158, 170)
Donegal	− 2.14 (− 3.95, − 0.33)	0.021	186 (176, 195)	140 (131, 149)
Dublin	−1.56 (− 2.39, − 0.73)	< 0.001	180 (175, 184)	138 (134, 142)
Galway	− 1.51 (− 3.14, 0.12)	0.070	189 (181, 198)	149 (140, 157)
Kerry	−2.04 (− 3.69, − 0.39)	0.015	198 (188, 208)	189 (178, 201)
Kildare	0.29 (− 1.79, 2.37)	0.785	188 (176, 200)	152 (141, 163)
Kilkenny	−0.3 (− 3.01, 2.41)	0.830	166 (153, 179)	145 (132, 159)
Laois	− 0.05 (− 3.07, 2.97)	0.973	183 (165, 201)	146 (131, 161)
Leitrim	0.98 (− 2.77, 4.72)	0.609	208 (185, 230)	171 (149, 193)
Limerick	− 0.39 (− 2.04, 1.26)	0.644	202 (191, 212)	179 (170, 189)
Longford	−0.06 (− 3.59, 3.48)	0.975	201 (181, 221)	168 (148, 187)
Louth	−2.48 (− 4.69, − 0.28)	0.027	173 (162, 184)	136 (125, 148)
Mayo	− 4.6 (− 6.31, − 2.88)	< 0.001	261 (249, 273)	155 (145, 165)
Meath	−1.38 (− 3.65, 0.88)	0.230	154 (143, 164)	131 (121, 141)
Monaghan	1.47 (−1.45, 4.4)	0.324	173 (159, 187)	164 (147, 180)
Offaly	−1.56 (− 4.36, 1.24)	0.274	200 (184, 216)	161 (145, 176)
Roscommon^a^	− 0.38 (− 2.99, 2.23)	0.773	147 (134, 159)	186 (165, 207)
Sligo	−3.31 (−6.12, − 0.5)	0.021	203 (187, 218)	136 (122, 150)
Tipperary	−1.12 (−2.98, 0.74)	0.237	201 (191, 210)	154 (144, 164)
Waterford	0.16 (−2.17, 2.5)	0.892	169 (158, 181)	137 (126, 149)
Westmeath	1.2 (−1.32, 3.72)	0.352	192 (177, 206)	163 (149, 176)
Wexford	−0.36 (−2.31, 1.59)	0.715	170 (159, 180)	159 (148, 169)
Wicklow	−0.82 (−3.04, 1.41)	0.472	192 (179, 204)	154 (142, 166)

^a^Roscommon result does not include 2011/2012

**Fig. 3:**
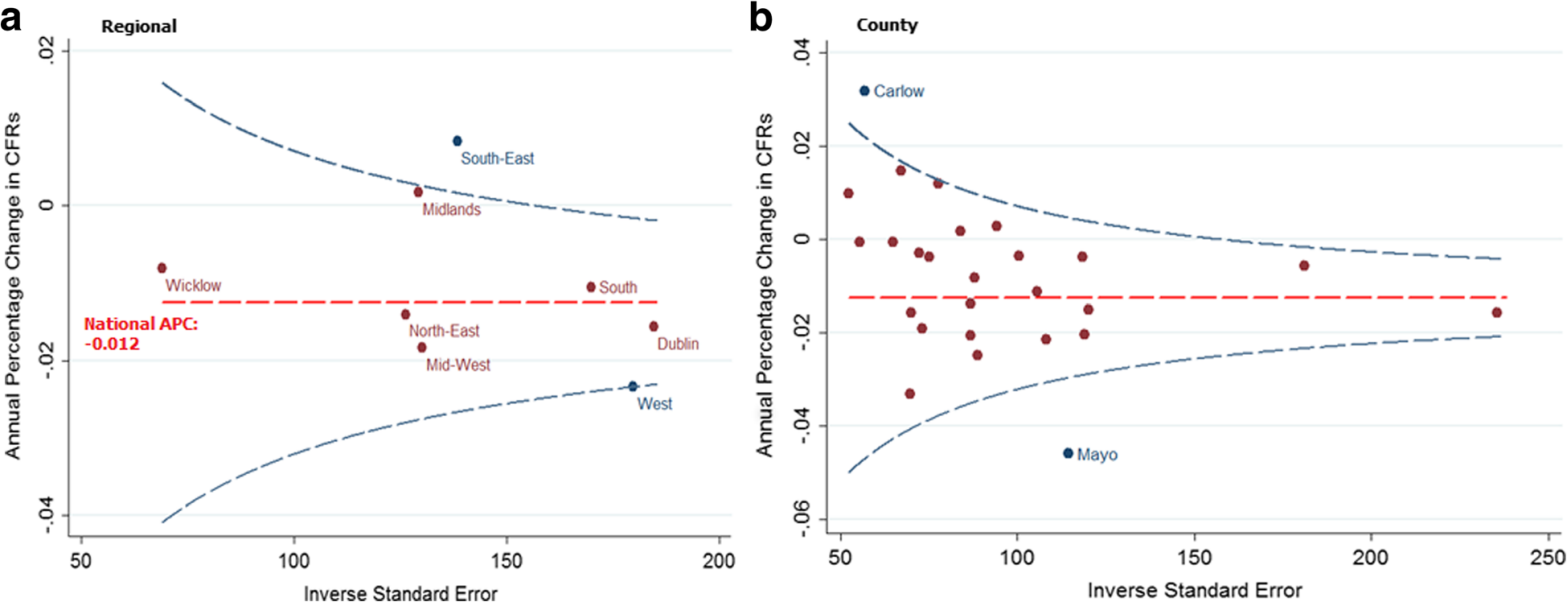
Annual percentage change (APC) in age and case-mix adjusted CFRs with a 95% control limit. The target is the national APC of − 1.2%

When compared to the national annual decrease (− 1.2%) for this period, two regions were found to have a statistically significant difference; the South-East had a lower rate of decrease, and the West had a higher rate of decrease (Fig. 3).

The county results identified six counties with significant decreases in their CFR trends (Donegal, Dublin, Kerry, Louth, Mayo, and Sligo) from 2007 to 2014 (Table 2). Carlow and Mayo were found to be outside of the 95% limit when compared to the national trend (Fig. 3).

### Cross sectional analysis of county CFRs in two time periods

**Fig. 4:**
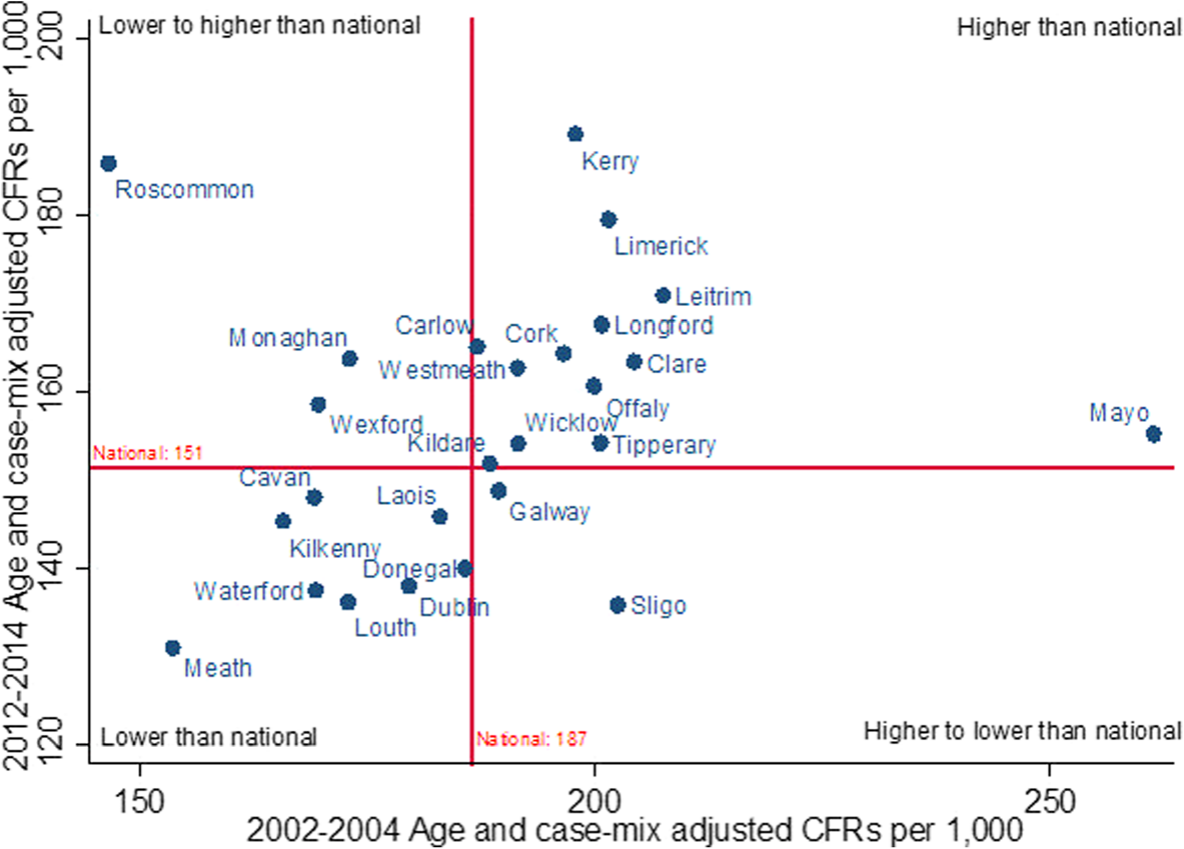
Comparison of CFRs for 2 time periods; 2002–2004 and 2012–2014

A comparison of county total CFR results in 2002–2004 and 2012–2014 to the respective national CFRs can be seen in Fig. 4, represented by the horizontal and vertical red lines, and Table 1.

The Spearman rank correlation of the CFRs found a statistically significant relationship between county results over the 2 periods (ρ = 0.40, *P* = 0.04). Three counties (Monaghan, Roscommon and Wexford) had a decline in their position with regards to the national result between periods, and two counties (Galway and Sligo) improved (Fig. 4). The two counties identified as significantly different from the national trend, Carlow and Mayo, were above the national result in both periods.

The coefficient of variation found that variability between county CFRs decreased in the two periods; 11.45 for 2002–2004 and 9.72 for 2012–2014.

## Discussion

### Summary of findings

There was a large decline nationally in case fatality from serious emergency conditions over the period 2002–2014. The continued decrease in case fatality, albeit at a slower rate from 2007, is a positive outcome at a national level. Pronounced changes for stroke and the ‘other’ group were seen, while the rate of decline for AMI and cardiac arrest did not slow. The number of events for acute myocardial infarction has consistently decreased during the period of study (see Additional file 4). This is despite changes in how the condition is defined, and continued improvements in its detection [13, 26].

Variability in outcomes persists at a regional level. Two of the eight regions showed a significant difference in total condition fatality when compared to the 2007–2014 national rate; the South-East improving slower, while the West improved faster. Variation at county level also demonstrates that within region results are not homogenous.

Cross-sectional analysis revealed many counties in the South-East, North-East and Dublin performed consistently better than the national CFR. These regions underwent limited attempts at reconfiguration. The South, Mid-West and Midlands regions were consistently worse than the national average CFR over the study period. Of these, the South and Mid-West regions underwent significant reconfiguration.

There is little evidence that identified changes in CFRs at a regional or county level were associated with the reconfiguration of services, such as the removal of emergency departments. For example, counties such as Monaghan and Roscommon, which experienced the closure of emergency departments, saw a decrease in their position relative to the national CFR over the two time periods (2002–2004 and 2012–2014) studied. However, the rate of CFR decrease in these counties was not statistically different from the national rate between 2007 and 2014, as per the constructed funnel plots.

### Interpretation

The findings from this research reveal a complex picture. Undoubtedly outcomes have improved over the period in question; however the national rate of improvement slowed in the most recent years. An argument can be made that mortality may be the last thing affected by system change. Clinical professionalism may limit any potentially negative consequences of such changes. Aspects of quality, safety and morbidity, including a delay in care and unrelieved pain, may be more likely to experience adverse effects.

The concentration of emergency care to specialist centres is intended to improve outcomes [11–14]. In the UK, for example, the reconfiguration of trauma care services led to a 60% improvement in the odds of surviving a major trauma over the period 2008–2014 [27]. The findings presented in this paper suggest that reconfiguration in Ireland, mainly implemented after 2006, has not resulted in improved outcomes, and has not altered long-term geographical differences between regions and counties. This may be due to poor resourcing and implementation of reconfiguration plans. It may also be due to long-term structural differences between geographical areas in social determinants of health such as rurality and deprivation. Any detailed cross sectional analysis of variations between counties would need to account for these characteristics. A study of mortality in England and Wales found that deprivation accounted for the majority of differences seen between urban and rural areas, with the exception of lung cancer, respiratory disease and accidents [19].

The period of study also saw improved clinical guidelines and documents of best practice, the establishment of offices of clinical audit, as well as the introduction of clinical care programmes for conditions such as stroke and AMI.

The National Stroke Programme launched in 2010 is considered to have substantially changed the level of specialised stroke care received by patients [28]. A 2015 national audit of stroke highlighted in-hospital improvements for stroke mortality, decreasing from 19 to 14% since 2008 [29].

One of the principal aims of this programme was the development of stroke units in all hospitals which accept stroke patients [28]. However, issues exist regarding the full implementation and staffing of these units. According to the audit, only 29% of patients were admitted directly to a stroke unit and almost 50% did not receive treatment in a unit during their stay in hospital [29]. Also, nearly a quarter of the hospitals providing acute stroke care did not meet the minimum standards of a stroke unit [29].

The goal of full national 24/7 thrombolysis has still not been achieved. It is currently supported through bypass protocols to larger tertiary hospitals when required, and the development of the Telemedicine Rapid Access for Stroke and Neurological Assessment (TRASNA). TRASNA allows doctors to provide consultations via video and supervise thrombolysis where necessary. Where implemented the rate of thrombolysed patients is 1 in every 3.5 patients, compared to 1 in 5 elsewhere [28]. However, delays have been experienced in the full roll out of this programme [28].

In terms of cardiac care, the Acute Coronary Syndromes (ACS) Programme was launched in 2012 [30]. This programme has supported the adoption of five 24/7 primary percutaneous coronary intervention (PCI) centres and one 9–5 Monday to Friday centre nationally [31]. Improvements have also been made to pre-hospital services for patients as a result of changes to pre-hospital emergency care council and ambulance protocols. It has subsequently been reported that the number of reperfused ST-Elevation Myocardial Infarction (STEMI) patients that receive PCI increased from 55% in 2011 to 94% in 2015 [31].

The impact of these condition specific service changes and other clinical programmes, together with higher level system changes, can be seen in the results of our analysis. The slowing of improvement, particularly for stroke, may now be a result of gains being harder to achieve as programmes start to focus on more complex changes. At a regional level, initial emergency care system resources and quality of care were not uniform and the implementation of reconfiguration differed widely across regions. Changes took place in the context of an initial period of national investment and growth, followed by an economic recession. Budgetary cuts were a contributing factor to the structural changes which resulted in the closure of emergency services. Restrictions on staff recruitment across emergency departments and ambulance services continue to be experienced to date. For instance, a review of the National Ambulance Service (NAS) in 2015 found that almost 300 additional staff would be required to cover best achievable performance [32], while the 2016 National Service Plan highlighted the continued gap between pre and post-recession employment in the acute hospital sector [33].

### Context of the literature

The restructuring of emergency services has been previously studied internationally, particularly with regard to the closure of rural emergency departments. Conflicting results have been found. Some studies [15, 34] have found a risk of higher mortality when distance to treatment is increased. In the UK, a study found that a 10-km increase in straight-line distance to treatment was associated with a 1% absolute increase in mortality [15]. Conversely, a study in the United States concluded that higher in-hospital mortality did not necessarily occur after the closure of a local emergency department [35]. It argued that where other appropriate services exist, the closure or reduction of certain services will not have a negative impact on in-hospital mortality outcomes [35]. However, remaining facilities must be adequately resourced and staffed to meet new demands [36].

### Strengths and limitations

A strength of this study is the shift from in-hospital mortality as the main measure of outcome. Using hospital mortality rates to predict the quality of hospital care can result in good or average hospitals being penalised [37]. Its continued use in outcome reporting [4–6] over-emphasises the concerns of providers, rather than the needs of the population. Case fatality constructed by area of residence allows analysis of outcomes for those who need to engage with the system, rather than focusing on outcomes from a specific service [23].

This study is subject to a number of limitations. Emergency admissions to private hospitals were not included in this analysis; private hospitals are not required to submit data to the hospital inpatient enquiry system (HIPE). Consequentially, case fatality ratio results reported may represent a maximum level; results for counties with a high level of private hospital usage may be lower than stated here. However, we estimate the impact of private hospital admission on our results is low due to a number of factors. First, there were only five small private emergency departments open in Ireland over the study period and many of those were not open for the full study period. Second, these hospitals generally worked on a 8 am-5 pm schedule, Monday to Friday [38, 39] and during our study period would not have operated a weekend service. Third, private emergency departments generally did not accept the most serious emergency conditions, such as major trauma and acute stroke, over the study period [38–40]. Fourth, serious emergency cases requiring ambulances were not taken to private emergency departments over the study period [38, 39].

Our analyses rely on the accuracy of the HIPE system for recording emergency admissions. A study by the Department of Health in 2013 has confirmed the robustness of the data available from HIPE, specifically as a tool for the development of indicators of quality of care in hospitals [41]. As a result, this data has formed the basis of the National Healthcare Quality Reporting System annual reports [4–6] and such use is in line with the analysis produced within this study. However, within our analysis particular caution should be used when interpreting results for County Roscommon. Due to the absence of a HIPE coder for a period spanning part of 2011–2012 in Roscommon County Hospital, the accuracy of coding is limited for much of the county’s patient population.

The primary aim of this study is the evaluation of major system change. Reconfiguration of such a scale is likely to lead to improved results for certain conditions, but the deterioration of results for others. Therefore, to assess the overall impact on the system, the focus is necessarily on aggregated higher level data. The analysis of patients, or each condition, at an individual level is of limited benefit.

Cautions should be taken when using county level data in understanding change in complex, multi-factor situations. However, it is important to note that any lower level analysis is restricted in Ireland due to lack of access to more detailed data. Access to admissions data through the hospital admissions system is limited to county level. Similarly, personal individual level mortality data is unavailable from the Central Statistics Office due to concerns of identifiability.

Ireland also differs from many other European countries in that it does not have a unique patient identifier. This restricts the ability to link individuals to admissions and subsequent death for a specific condition. Therefore, analysis was limited to the ratio of deaths to cases in a year, as opposed to the rate of deaths per cases. There are measures underway as of 2014 to introduce a National Register of Individual Health Identifiers [42].

### Policy implications

There is currently no independent routine health planning on behalf of populations in Ireland. The majority of planning is done by, or on the behalf of, the provider, the health service executive (HSE). Such planning is primarily based on once off national reports, as previously outlined [7–10], which focus on the performance of the provider. This study provides a counterpoint to such reports, and aims to refocus attention to how well populations are served.

Our findings show that changes to the national CFR trend coincided with a period of recession in Ireland. With additional budget allocations as of 2015 [33], further monitoring will determine if there are future improvements to CFRs. Additionally, policies of reconfiguration do not appear to have significantly influenced CFRs. Continued observation will determine if on-going implementation of these policies also result in greater improvements. It may also be argued that much of the variance in case-fatality can be explained by non-health system factors such as deprivation and rurality [19, 43, 44], which have not been included in our model and merit further investigation.

## Conclusion

National outcomes for serious emergency conditions have improved over the period 2002–2014 in Ireland. However, a slowing of the rate of improvement since 2007 coincided with a period of economic contraction. Changes to fatality trends varied by condition; therefore, results cannot be solely attributed to recessionary factors.

The impact of individual clinical programmes, and subsequent system changes to services such as stroke units and PCI centres, must also be considered.

Persistent geographical variation in case fatality remains despite attempts to reconfigure regional services. A distinct pattern cannot be identified between regions and counties that undertook substantial reconfiguration of emergency services and those that did not. Further research on the role of rurality and deprivation in driving outcome and process variation, the role of regional variation in resources, and the extent to which reconfiguration plans were fully implemented, is planned by the SIREN research collaboration.

## Additional files

Additional file 1: Table S1 Reconfiguration of emergency care systems in Ireland. (PDF 294 kb)

Additional file 2: Table S2 Basket of Emergency Conditions by ICD9 and ICD10. (PDF 194 kb)

Additional file 3: Table S3 Deaths from emergency conditions used in the indicator analysis by year excluding Roscommon. (PDF 216 kb)

Additional file 4: Table S4 Events from emergency conditions used in the indicator analysis by year excluding Roscommon. (PDF 218 kb)

Additional file 5: Table S5 Number of deaths and survivors by emergency conditions 2002–2014. (PDF 201 kb)

## Abbreviations

ACS: Acute coronary syndromes
AMI: Acute myocardial infarction
APC: Annual percentage change
CFR: Case fatality ratio
CI: Confidence interval
CSO: Central statistics office
HIPE: Hospital inpatient enquiry
HPO: Health pricing office
HRB: Health research board
ICD: International statistical classification of diseases and related health problems
NAS: National ambulance service
OECD: Organisation for economic co-operation and development
PCI: Percutaneous coronary intervention
SIREN: Study of the impact of reconfiguration on emergency and urgent care networks
STEMI: ST-elevation myocardial infarction
TRASNA: Telemedicine rapid access for stroke and neurological assessment
UK: United Kingdom
