# Supplementary material for: Case fatality ratios for serious emergency conditions in the Republic of Ireland: a longitudinal investigation of trends over the period 2002–2014 using joinpoint analysis
Source: BMC Health Serv Res. 2018 Jun 19;18:474. doi: 10.1186/s12913-018-3260-1 (PMC6006987; doi:10.1186/s12913-018-3260-1)
Supplement: Supplementary file 1 — Table S1 Reconfiguration of emergency care systems in Ireland. (PDF 294 kb) [file 12913_2018_3260_MOESM1_ESM.pdf]

Additional file 1: Table 1 Reconfiguration of emergency care systems in Ireland

Region	Characteristics	Summary of Regional Change
Significant Reconfiguration		
**Region**	**Characteristics**	**Summary of Regional Change**
**South**<br>(Cork and Kerry)	Population: 663,176<br>Area (km^2): 12,161	**Regional Reconfiguration**<br>• Region-specific reconfiguration plan largely implemented, beginning 2012-2013<br>• Region-wide clinical governance structures established<br>• Single general practice (GP) out of hours co-operative<br>**Emergency Department Services Reconfiguration**<br>• Acute stroke, coronary and major trauma care provided at hub in Cork [Cork University Hospital] with support of ambulance protocols and outlying centres [Kerry: University Hospital Kerry; Cork: Bantry General Hospital]<br>• Two EDs reconfigured to local injury units [Cork: Mallow General Hospital (2013) and Bantry General Hospital (2013)]<br>• One emergency department (ED) closed [Cork: South Infirmary Hospital (2012)]
**Mid-West**<br>(Limerick, Clare and Tipperary North)	Population: 378,210<br>Area (km^2): 8,252	**Regional Reconfiguration**<br>• Region-specific reconfiguration plan largely implemented, 2009 - 2013<br>• Ambulance bypass protocols and region-wide clinical directorates established<br>• Single GP out of hours co-operative<br>**Emergency Department Services Reconfiguration**<br>• All emergency care centralised to one hospital [Limerick: University Hospital Limerick]<br>• Two EDs reconfigured to local injury units [Clare: Ennis Hospital (2009); Tipperary North: Nenagh Hospital (2009)]
Some Reconfiguration		
**Region**	**Characteristics**	**Summary of Regional Change**
**West**<br>(Galway, Roscommon, Mayo, Leitrim, Sligo, Donegal)	Population: 702,966<br>Area (km^2): 22,649	**Regional Reconfiguration**<br>• Clinical directorates established across the region<br>• Several out of hours GP co-operatives<br>**Emergency Department Services Reconfiguration**<br>• Single hub for acute coronary and major trauma care [Galway: University Hospital Galway] with major trauma support services provided at other centres [Mayo: Mayo University Hospital; Donegal: Letterkenny University Hospital; Sligo: Sligo University Hospital]. Acute stroke care at all centres, excluding Roscommon General Hospital<br>• One ED reconfigured to local injury unit [Roscommon: Roscommon General Hospital (2011)]<br>• No ED in Leitrim
**North East**<br>(Cavan, Meath, Louth and Monaghan)	Population: 440,211<br>Area (km^2): 6,395	**Regional Reconfiguration**<br>• Region-specific reconfiguration plan partly implemented from 2006 – 2010<br>• Limited regional clinical governance<br>• Roll-out of general practitioner (GP) out of hours care<br>**Emergency Department Services Reconfiguration**<br>• Some centralisation of trauma, acute stroke and coronary care [Cavan: Cavan General Hospital; Louth: Our Lady of Lourdes Drogheda] with

		rehab support in other hospitals<br>• Dublin North [Mater Hospital] is the percutaneous coronary intervention (PCI) centre with supporting ambulance protocols<br>• Two emergency departments reconfigured to local injury units [Louth:Louth County Hospital (2010); Monaghan: Monaghan General Hospital (2009)]
**South East**<br>(Carlow, Kilkenny, Wexford, Waterford and Tipperary South)	Population: 497,305<br>Area (km^2): 9,451	**Regional Reconfiguration**<br>• Informal clinical network with shared regional rota for emergency medicine consultants<br>• Single GP out of hours co-operative.<br>**Emergency Department Services Reconfiguration**<br>• Designated hub for major trauma, and acute coronary care [Waterford: Waterford Regional Hospital – PCI centre supported out of hours by Cork] with ambulance bypass protocols<br>• Acute stroke care available at all 4 hospitals<br>• No ED in Carlow
**Dublin South**<br>(Dublin South City, Dun Laoghaire Rathdown, Wicklow)	Population: 563,560<br>Area (km^2): 2,168	**Regional Reconfiguration**<br>• Multiple out of hours GP co-operatives<br>**Emergency Department Services Reconfiguration**<br>• Centralisation of acute stroke, coronary and trauma care to two hospitals (both in Dublin South City) but limited differentiation and integration between both<br>• One ED reconfigured to local injury unit [Dun Laoghaire Rathdown: St Columcille's Hospital (2013)]<br>• One ED with reduced hours [Dun Laoghaire Rathdown: St Michaels (2003)]<br>• No ED in Wicklow
Little reconfiguration		
**Region**	**Characteristics**	**Summary of Regional Change**
**Dublin North East**<br>(Fingal, Dublin North City)	Population: 578,317<br>Area (km^2): 532	**Regional Reconfiguration**<br>• No major changes<br>• Out of hours GP co-operative established<br>**Emergency Department Services Reconfiguration**<br>• Three large emergency departments with limited governance integration and differentiation of services. PCI Centre established [Dublin North: Mater Hospital]
**Dublin Midlands**<br>(Dublin South, Longford, Westmeath, Laois, Offaly, Kildare)	Population: 761,324<br>Area (km^2): 8,442	**Regional Reconfiguration**<br>• Limited integration of clinical governance<br>• Several out of hours GP co-operatives operating<br>**Emergency Department Services Reconfiguration**<br>• Centralisation of acute stroke [Kildare: Naas General Hospital; Westmeath: Midlands Regional Hospital Mullingar, and Dublin South: Tallaght Hospital) coronary care [Dublin South: Tallaght Hospital] and trauma [Offaly: Midland Regional Hospital Tullamore; Dublin South: Tallaght Hospital) at several hospitals, supported by ambulance bypass protocols<br>• No ED in Longford
