# Supplementary material for: Case fatality ratios for serious emergency conditions in the Republic of Ireland: a longitudinal investigation of trends over the period 2002–2014 using joinpoint analysis
Source: BMC Health Serv Res. 2018 Jun 19;18:474. doi: 10.1186/s12913-018-3260-1 (PMC6006987; doi:10.1186/s12913-018-3260-1)
Supplement: Supplementary file 2 — Table S2 Basket of Emergency Conditions by ICD9 and ICD10. (PDF 194 kb) [file 12913_2018_3260_MOESM2_ESM.pdf]

Additional file 2: Table 2 Basket of Emergency Conditions by ICD-9 and ICD-10

Emergency Conditions		
	**ICD-9**<br>**HIPE (2002-2004)/ CSO (2002-2006)**	**ICD-10**<br>**HIPE (2005-2012)/CSO (2007-2012)**
**STROKE**		
Stroke	432.9; 431; 433; 434; 436	I61; I63; I64; I62.9
**AMI and CA**		
Acute Myocardial Infarction	410	I21; I22; I23
Cardiac Arrest	427.5	I46; I46.9
**OTHER**		
Acute Heart Failure	428	I50
Anaphylaxis	995.0; 995.6	T78·0; T78.2; T80.5;T88.6
Asphyxiation	799.0; 994.7	R09.0; T71
Asthma	493.0; 493.1; 493.8; 493.9	J45; J46
Falls under 75	E880 –E888	W00 - W19
Fractured Neck of Femur	820; 821	S72
Meningitis	320- 322; 036; 027	G00 - G03; A32; A39
Pregnancy	630 – 679	O00 - O99
Road Traffic Accident	E800 - E807; E810- E829	V0-V7; V80.2-V80·5; V82.1; V83.0-V83.3; V84.0–V84.3; V85.0–V85.3; V86.0–V86.3; V87.0–V87.8; V89.2
Ruptured Aortic Aneurysm	441.0; 441.1; 441.3; 441.5; 441.6	I71.0; I71.1; I71.3; I71.5; I71.8
Self-Harm	E950-E959	X6- X84
Septic Shock	038	A40; A41
Serious Head Injury	800-804; 830; 850-854; 870- 874; 900; 925; 940-941; 950- 951	S02-S09
