# Supplementary material for: Case fatality ratios for serious emergency conditions in the Republic of Ireland: a longitudinal investigation of trends over the period 2002–2014 using joinpoint analysis
Source: BMC Health Serv Res. 2018 Jun 19;18:474. doi: 10.1186/s12913-018-3260-1 (PMC6006987; doi:10.1186/s12913-018-3260-1)
Supplement: Supplementary file 3 — Table S3 Deaths from emergency conditions used in the indicator analysis by year excluding Roscommon. (PDF 216 kb) [file 12913_2018_3260_MOESM3_ESM.pdf]

Additional File 3: Table 3 Deaths from emergency conditions used in the indicator analysis by year (*excluding Roscommon)

Condition	2002	2003	2004	2005	2006	2007	2008	2009	2010	2011*	2012*	2013	2014	Total (Col%)
**Stroke**														
Stroke	1967	1808	1625	1535	1449	1528	1500	1480	1599	1491	1478	1534	1489	**20483 25.28%**
**AMI and CA**														
Acute Myocardial Infarction and Cardiac Arrest	3686	3242	3195	2938	2976	2762	2660	2458	2369	2086	2234	2122	1867	**34595 42.70%**
**Other**														
Acute Heart Failure	698	660	606	536	556	511	496	438	558	484	491	555	495	**7084 8.74%**
Anaphylaxis	-	-	1	-	-	2	-	1	1	-	-	1	-	**6 0.01%**
Asphyxiation	253	281	305	325	334	315	365	405	386	426	423	373	379	**4570 5.64%**
Asthma	70	64	76	62	52	61	52	53	44	53	39	48	40	**714 0.88%**
Falls <75	30	29	29	37	37	34	26	31	33	36	27	18	25	**392 0.48%**
Fractured Neck of Femur	167	172	139	154	134	111	104	87	87	107	96	90	129	**1577 1.95%**
Meningitis	23	26	13	22	13	18	20	12	12	8	12	9	7	**195 0.24%**
Pregnancy and Birth Related	5	-	1	1	1	2	3	3	1	2	2	3	1	**25 0.03%**
Road Traffic Accident NECs	249	214	211	214	214	181	139	114	103	95	88	85	93	**2000 2.47%**
Ruptured Aortic Aneurysm	235	243	200	224	243	254	228	215	225	221	221	238	187	**2934 3.62%**
Self-Harm	214	205	174	174	130	156	144	158	127	137	124	128	125	**1996 2.46%**
Septic Shock	57	40	38	50	44	85	67	78	100	98	81	91	141	**970 1.20%**
Serious Head Injury	324	288	287	322	271	269	269	255	247	255	227	233	227	**3474 4.29%**
**Other: Subtotal**	**2325**	**2222**	**2080**	**2121**	**2029**	**1999**	**1913**	**1850**	**1924**	**1922**	**1831**	**1872**	**1849**	**25937 32.02%**
**Total**	**7978 9.85%**	**7272 8.98%**	**6900 8.52%**	**6594 8.14%**	**6454 7.97%**	**6289 7.76%**	**6073 7.50%**	**5788 7.14%**	**5892 7.27%**	**5499 6.79%**	**5543 6.84%**	**5528 6.82%**	**5205 6.42%**	**81015 100%**
