# Supplementary material for: Case fatality ratios for serious emergency conditions in the Republic of Ireland: a longitudinal investigation of trends over the period 2002–2014 using joinpoint analysis
Source: BMC Health Serv Res. 2018 Jun 19;18:474. doi: 10.1186/s12913-018-3260-1 (PMC6006987; doi:10.1186/s12913-018-3260-1)
Supplement: Supplementary file 4 — Table S4 Events from emergency conditions used in the indicator analysis by year excluding Roscommon. (PDF 218 kb) [file 12913_2018_3260_MOESM4_ESM.pdf]

Additional file 4: Table 4 Events (deaths + emergency admissions discharged alive after a minimum 2 day length of stay) for emergency conditions used in the indicator analysis by year (*excluding Roscommon)

Condition	2002	2003	2004	2005	2006	2007	2008	2009	2010	2011*	2012*	2013	2014	Total (Col%)
**Stroke**														
Stroke	5861	5768	5595	5281	5342	5405	5465	5648	5786	5526	5535	5612	5727	**72551**<br>**14.74%**
**AMI and CA**														
Acute Myocardial Infarction and Cardiac Arrest	7738	7177	7297	7194	7295	7130	6950	6690	6494	6234	6391	6319	6081	**88990**<br>**18.08%**
**Other SECs**														
Acute Heart Failure	5028	4695	4546	4381	4401	4366	4211	4274	4327	3955	4116	4294	4253	**56847**<br>**11.55%**
Anaphylaxis	48	36	30	41	32	25	34	30	34	21	36	32	35	**434**<br>**0.09%**
Asphyxiation	271	300	313	357	360	335	390	442	422	461	457	404	418	**4930**<br>**14.74%**
Asthma	2580	2771	2570	2512	2425	2104	2086	1834	1775	1632	1861	1597	1789	**27536**<br>**5.60%**
Falls <75	6266	6186	6187	5858	6282	6552	6318	6168	6779	5403	5292	5340	5352	**77983**<br>**15.85%**
Fractured Neck of Femur	3674	3796	3742	3702	3827	3694	3837	3781	3895	3630	3554	3750	3940	**48795**<br>**9.91%**
Meningitis	350	365	322	314	344	294	283	287	225	215	191	233	196	**3619**<br>**0.74%**
Pregnancy and Birth Related	54	57	29	54	82	52	75	73	60	37	11	4	3	**591**<br>**0.12%**
Road Traffic Accident NECs	2993	2676	2673	2269	2235	2226	2029	1779	1573	1378	1317	1295	1392	**25835**<br>**5.25%**
Ruptured Aortic Aneurysm	283	318	270	294	311	327	299	294	316	286	308	338	286	**3930**<br>**0.80%**
Self-Harm	1906	1845	1654	1632	1630	1703	1706	1556	1551	1421	1466	1382	1393	**20845**<br>**4.24%**
Septic Shock	753	751	787	816	869	972	1036	1108	1207	1163	1449	1598	1956	**14465**<br>**2.94%**
Serious Head Injury	3840	3701	3818	3878	3646	3778	3472	3609	3350	3106	2874	2814	2915	**44801**<br>**9.10%**
**Other: Subtotal**	**28046**	**27470**	**26941**	**26108**	**26444**	**26428**	**25776**	**25235**	**25514**	**22708**	**22932**	**23081**	**23928**	**330611**<br>**67.18%**
**Total**	**41645**<br>**8.46%**	**40415**<br>**8.21%**	**39833**<br>**8.09%**	**38583**<br>**7.84%**	**39081**<br>**7.94%**	**38963**<br>**7.92%**	**38191**<br>**7.76%**	**37573**<br>**7.63%**	**37794**<br>**7.68%**	**34468**<br>**7.00%**	**34858**<br>**7.08%**	**35012**<br>**7.11%**	**35736**<br>**7.26%**	**492152**<br>**100%**
