# Supplementary material for: Case fatality ratios for serious emergency conditions in the Republic of Ireland: a longitudinal investigation of trends over the period 2002–2014 using joinpoint analysis
Source: BMC Health Serv Res. 2018 Jun 19;18:474. doi: 10.1186/s12913-018-3260-1 (PMC6006987; doi:10.1186/s12913-018-3260-1)
Supplement: Supplementary file 5 — Table S5 Number of deaths and survivors by emergency conditions 2002–2014. (PDF 201 kb) [file 12913_2018_3260_MOESM5_ESM.pdf]

Additional file 5: Table 5 Number (%) of deaths and survivors by emergency conditions, 2002-2014

Condition		Status		Total
		Alive	Dead	
**Stroke**				
Stroke	Count	52068	20483	72551
	% within condition	71.77%	28.23%	100.00%
**AMI and CA**				
Acute Myocardial Infarction and Cardiac Arrest	Count	54395	34595	88990
	% within condition	61.12%	38.88%	100.00%
**Other**				
Acute Heart Failure	Count	49763	7084	56847
	% within condition	87.54%	12.46%	100.00%
Anaphylaxis	Count	428	6	434
	% within condition	98.62%	1.38%	100.00%
Asphyxiation	Count	360	4570	4930
	% within condition	7.30%	92.70%	100.00%
Asthma	Count	26822	714	27536
	% within condition	97.41%	2.59%	100.00%
Falls <75	Count	77591	392	77983
	% within condition	99.50%	0.50%	100.00%
Fractured Neck of Femur	Count	47218	1577	48795
	% within condition	96.77%	3.23%	100.00%
Meningitis	Count	3424	195	3619
	% within condition	94.61%	5.39%	100.00%
Pregnancy and Birth Related	Count	566	25	591
	% within condition	95.77%	4.23%	100.00%
Road Traffic Accident	Count	23835	2000	25835
	% within condition	92.26%	7.74%	100.00%
Ruptured Aortic Aneurysm	Count	996	2934	3930
	% within condition	25.34%	74.66%	100.00%
Self-Harm	Count	18849	1996	20845
	% within condition	90.42%	9.58%	100.00%
Septic Shock	Count	13495	970	14465
	% within condition	93.29%	6.71%	100.00%
Serious Head Injury	Count	41327	3474	44801
	% within condition	92.25%	7.75%	100.00%
**Total**	**Count**	**411137**	**81015**	**492152**
	**% within condition**	**83.54%**	**16.46%**	**100.00%**
